# Is the Relationship between Prenatal Exposure to PCB-153 and Decreased Birth Weight Attributable to Pharmacokinetics?

**DOI:** 10.1289/ehp.1206457

**Published:** 2013-08-09

**Authors:** Marc-André Verner, Robin McDougall, Anders Glynn, Melvin E. Andersen, Harvey J. Clewell, Matthew P. Longnecker

**Affiliations:** 1 Channing Division of Network Medicine, Brigham and Women’s Hospital, Harvard Medical School, Boston, Massachusetts, USA; 2 Institute of Environmental Medicine, Karolinska Institutet, Stockholm, Sweden; 3 University of Ontario Institute of Technology, Oshawa, Ontario, Canada; 4 Swedish National Food Agency, Uppsala, Sweden; 5 Department of Environmental Toxicology, Evolutionary Biology Center, Uppsala University, Uppsala, Sweden; 6 The Hamner Institutes for Health Sciences, Research Triangle Park, North Carolina, USA; 7 Epidemiology Branch, National Institute of Environmental Health Sciences, National Institutes of Health, Department of Health and Human Services, Research Triangle Park, North Carolina, USA

## Abstract

Background: A recent meta-analysis based on data from > 7,000 pregnancies reported an association between prenatal polychlorinated biphenyl (PCB)–153 exposure and reduced birth weight. Gestational weight gain, which is associated negatively with PCB levels in maternal and cord blood, and positively with birth weight, could substantially confound this association.

Objective: We sought to estimate the influence of gestational weight gain on the association between PCB-153 exposure and birth weight using a pharmacokinetic model.

Methods: We modified a recently published pharmacokinetic model and ran Monte Carlo simulations accounting for variability in physiologic parameters and their correlations. We evaluated the pharmacokinetic model by comparing simulated plasma PCB-153 levels during pregnancy to serial measurements in 10 pregnant women from another study population. We estimated the association between simulated plasma PCB-153 levels and birth weight using linear regression models.

Results: The plasma PCB-153 level profiles generated with the pharmacokinetic model were comparable to measured levels in 10 pregnant women. We estimated a 118-g decrease in birth weight (95% CI: –129, –106 g) for each 1-μg/L increase in simulated cord plasma PCB-153, compared with the 150-g decrease estimated based on the previous meta-analysis. The estimated decrease in birth weight was reduced to –6 g (95% CI: –18, 6 g) when adjusted for simulated gestational weight gain.

Conclusion: Our findings suggest that associations previously noted between PCB levels and birth weight may be attributable to confounding by maternal weight gain during pregnancy.

Citation: Verner MA, McDougall R, Glynn A, Andersen ME, Clewell HJ III, Longnecker MP. 2013. Is the relationship between prenatal exposure to PCB-153 and decreased birth weight attributable to pharmacokinetics? Environ Health Perspect 121:1219–1224; http://dx.doi.org/10.1289/ehp.1206457

## Introduction

Cord serum levels of PCB-153, a highly persistent polychlorinated biphenyl (PCB) congener, were recently reported to be associated with lower birth weight in a meta-analysis of data from > 7,000 pregnancies (Govarts et al. 2012). In the meta-analysis, the overall median concentration of PCB-153 in cord serum was 0.14 μg/L, and the summary estimate suggested a 150-g decrease in birth weight with each 1-μg/L increase in PCB-153. In light of the evidence that reduced birth weight is related to health and mortality in later life, these findings may be of particular importance for public health. However, because gestational weight gain is negatively associated with PCB levels in maternal and cord blood, and positively associated with birth weight, the association may be confounded by changes in physiology and pharmacokinetics during pregnancy rather than reflecting a causal effect of PCB exposure.

**Figure 1:**
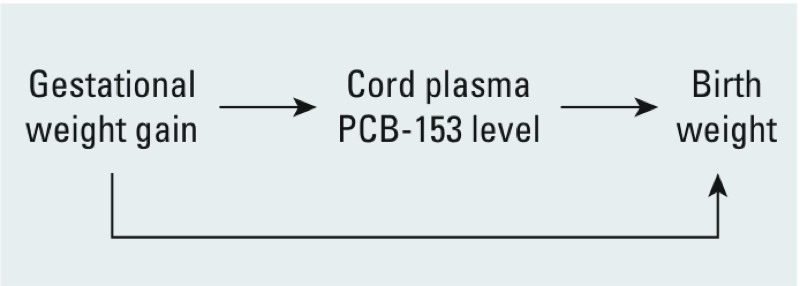
Conceptual diagram of the association between cord plasma PCB-153 levels and birth weight.

PCBs are highly lipophilic and consequently sequester mainly in body lipids. Among women whose weight gain during pregnancy is within the range recommended by the Institute of Medicine, about 30% of that weight gain is attributable to an increase in body lipids (Butte et al. 2003). For example, a 14.4-kg weight gain during pregnancy was associated with an average 4.1-kg increase in body fat (Butte et al. 2003). In the same study, gestational weight gain accounted for 58% of the variability in fat-mass gain. Fat gain during pregnancy increases the volume of distribution of lipophilic chemicals and, as a result, dilutes PCB levels in blood lipids (Glynn et al. 2007). On the other hand, gestational weight gain is positively associated with birth weight (Siega-Riz et al. 2009), making this variable a potential confounder of the exposure–outcome association (Figure 1). The extent to which the association between prenatal exposure to PCBs and birth weight is attributable to confounding by gestational weight gain has not been adequately considered.

We conducted this study to evaluate the influence of maternal and fetal physiology and pharmacokinetics on the association between prenatal exposure to PCB-153 and birth weight that was reported in the meta-analysis of Govarts et al. (2012). A previously published pharmacokinetic model was used to simulate changes in PCB-153 levels during pregnancy while accounting for time-dependent changes in physiology, weight gain, and body lipids. PCB-153 was used as a proxy for exposure to a mixture of PCB congeners because of its long half-life (Ritter et al. 2011) and its correlation with other congeners in biologic matrices (Gladen et al. 1999; Muckle et al. 2001).

## Methods

We assessed the influence of pharmacokinetics on the PCB–birth weight relationship with a two-step approach. First, we modified a pharmacokinetic model (Verner et al. 2013) to run Monte Carlo simulations where exposure and physiologic characteristics were varied to generate realistic population data. Next, we used the simulated population data in linear regression analyses to estimate the association between simulated PCB-153 concentrations in maternal or cord plasma and birth weight.

**Figure 2:**
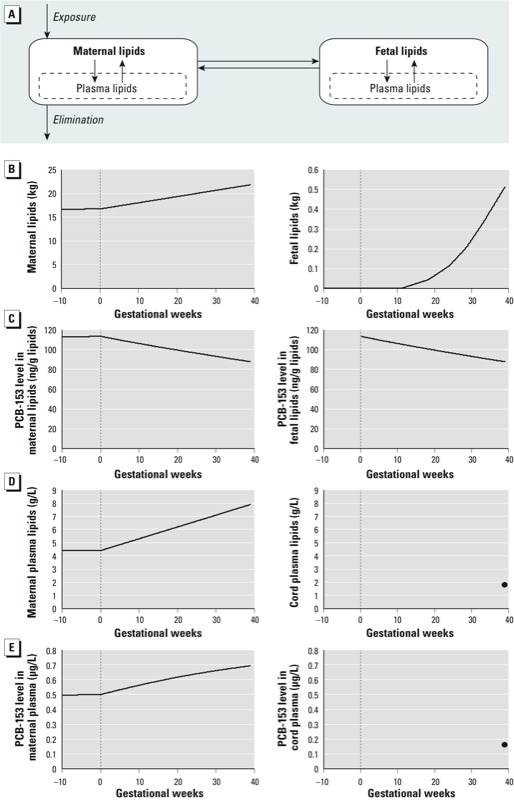
Pharmacokinetic model with homogeneous distribution of PCB‑153 across maternal and fetal lipids (*A*). An example of simulated lipid and PCB‑153 profiles is presented in *B–E* for a woman with a pre­pregnancy body weight of 60 kg, a gestational weight gain of 12.5 kg, and a 0.5‑μg/L plasma PCB-153 level at conception giving birth to a 3.4‑kg girl at 25 years of age: (*B*) amount of maternal and fetal lipids; (*C*) PCB‑153 levels in maternal and fetal lipids; (*D*) lipid content of maternal and cord plasma; (*E*) PCB-153 levels in maternal and cord plasma. The vertical dotted lines represent the time of conception. Because cord plasma lipid and PCB-153 levels were estimated only at delivery, single values (dots at 39 gestational weeks) are represented (*D,E*).

*The pharmacokinetic model*. The two-compartment pharmacokinetic model we used in this study is detailed by Verner et al. (2013). We opted for this simple model instead of the more complex physiologically based pharmacokinetic (PBPK) model presented by Verner et al. (2009) because modifications to model variations in pregnancy-related parameters, including gestational weight gain, were straightforward, and it requires less computation time. Briefly, this model simulates lifetime exposure and bioaccumulation in the mother and the fetus. Because PCB-153 partitions almost exclusively in lipids, only the lipid fraction of maternal and fetal tissues were represented in the model (Figure 2A). Maternal weight profiles from birth until conception were based on a normal growth curve (Kuczmarski et al. 2000) adjusted for prepregnancy body weight. Corresponding changes in body lipids were estimated based on body weight profiles and age-specific percentages of body fat (International Commission on Radiological Protection 2002). The increase in maternal lipids during pregnancy was estimated based on gestational weight gain as described below. We modeled changes in fetal lipid mass as a function of a normal growth profile (Guihard-Costa et al. 1995) adjusted to match birth weight and the percentage of lipids at birth (Enzi et al. 1981). An example of maternal and fetal lipid profiles during pregnancy is shown in Figure 2B. We assumed that PCB-153 distributes freely and homogeneously in maternal and fetal lipid compartments, including plasma lipids. PCB-153 elimination was parameterized assuming a half-life of 14.4 years (Ritter et al. 2011) and that elimination was independent of coexposures to other environmental contaminants. The complete model code and script are provided in Supplemental Material.

In their meta-analysis, Govarts et al. (2012) estimated the association between birth weight and PCB-153 levels in cord serum (wet-weight basis). To enable comparison with their results, we expressed lipid-basis levels from the pharmacokinetic model as wet-weight levels in maternal or cord plasma. We assumed that plasma and serum PCB-153 levels were equivalent because fibrinogen, the primary constituent that differs between serum and plasma, is unlikely to affect PCB-153 levels substantially. Maternal and cord plasma levels were derived by multiplying simulated PCB-153 levels in lipids by the assumed concentration of lipids in plasma:

Plasma PCB-153 (μg/L) = Lipid PCB-153 (μg/g lipids) *×* Plasma lipids (g/L). [1]

On average, maternal plasma lipids increase linearly during pregnancy, from 4.4 g/L at conception to 7.9 g/L at delivery [we averaged total plasma lipid levels during each trimester from data of from Aurell and Cramer (1966), Chiang et al. (1995), Mazurkiewicz et al. (1994), and van Stiphout et al. (1987)]. We assumed a cord plasma lipid concentration of 1.8 g/L (Denkins et al. 2000) to calculate cord plasma PCB-153 levels from fetal lipid PCB-153 at delivery. Average profiles of maternal and cord plasma lipids are depicted in Figure 2D. An example of maternal plasma PCB-153 profile and cord plasma level at delivery is shown in Figure 2E.

*Monte Carlo simulations*. Each Monte Carlo iteration represented an individual to whom physiologic parameters and plasma PCB-153 level at conception were assigned (Table 1). Parameters that remained constant for a given iteration (age at delivery, prepregnancy body weight, gestational weight gain, PCB-153 half-life, child sex, cord plasma lipid concentration, and plasma PCB-153 level at conception) were sampled from published distributions (Table 1). We varied parameters that fluctuate within individual iterations (maternal and fetal body lipids and maternal plasma lipid concentration) by multiplying average values by a multiplier sampled from a distribution centered around one where the standard deviation is equal to the reported coefficient of variation (Table 1). For example, maternal plasma lipids were calculated as follows:

**Table 1:** Parameter distributions used in the Monte Carlo simulations.

Parameter	Distribution	Mean ± SD	Minimum	Maximum	Reference
Mother
Age at delivery (years)	Normal	24.3 ± 6.1	15	41	NIH 2011^*a*^
Pre­pregnancy body weight (kg)	Normal	59.0 ± 11.6	25	163	NIH 2011
Gestational weight gain (kg)	Normal	10.3 ± 4.7	0	44	NIH 2011
Maternal lipids multiplier^*b*^	Normal	1.00 ± 0.16	0.68	1.32	Borrud et al. 2010
Plasma lipid multiplier^*b*^	Normal	1.00 ± 0.13	0.72	1.34	Glynn et al. 2011
Half-life PCB-153 (years)	Normal	14.4 ± 2.2	10.0	18.8	Ritter et al. 2011
Residual lipid gain (kg)	Normal	0.0 ± 2.7	–5.4	5.4	Butte et al. 2003
Infant
Sex	Bernoulli (*p *= 0.5)				—
Fetal lipids multiplier^*b*^	Normal	1.00 ± 0.17	0.66	1.34	Enzi et al. 1981
Cord plasma lipids (g/L)	Normal	1.83 ± 0. 24	1.35	2.31	Denkins et al. 2000
Residual birth weight (kg)	Normal	0.0 ± 0.5	–2.8	2.2	NIH 2011
Exposure
Maternal plasma PCB-153 at conception (ug/L)	Lognormal	0.487 ± 0.412^*c*^	0.001	10.000	Govarts et al. (2012)^*d*^
^***a***^National Institutes of Health; the Collaborative Perinatal Project. ^***b***^Parameters fluctuating within individual simulations (e.g., age-related changes in the amount of maternal lipids) were varied using a multiplier. ^***c***^Arithmetic mean ± SD. ^***d***^The distribution of maternal plasma PCB-153 levels at conception was adjusted to yield a distribution of cord plasma levels similar to that reported by Govarts et al. (2012), i.e., mean ± SD of 0.180 ± 0.157 μg/L.					

Maternal plasma lipids (g/L)*_i_* = Average maternal plasma lipid concentration *×* Maternal plasma lipid multiplier*_i_*, [2]

where *i* indexes “subject” (i.e., Monte Carlo iteration) and using the coefficient of variation calculated based on data from Glynn et al. (2011) as the SD of the multiplier distribution.

The distribution of maternal plasma PCB-153 level at conception was adjusted to yield a distribution of cord plasma levels similar to that reported in Govarts et al. (2012). We estimated the daily PCB-153 absorbed dose (micrograms per kilogram body weight) through an iterative optimization process so that exposure to this daily dose over the woman’s lifetime resulted in a simulated blood level at conception matching that sampled from the distribution. This approach can encompass exposure through different routes because it is based on internal levels.

We induced correlations between gestational weight gain and both birth weight and maternal lipid gain during pregnancy by reconstructing linear regressions derived from the Collaborative Perinatal Project data (National Institutes of Health 2011) or obtained from the article by Butte et al. (2003). At each Monte Carlo iteration, a gestational weight gain was sampled from the distribution defined in Table 1 and used to estimate birth weight and maternal lipid gain based on linear regression equations. We replicated the dispersion of residuals in these associations by adding a value sampled from the residual distributions (Table 1). Not only does this approach replicate the correlation coefficient, but it also accounts for the slope, therefore yielding realistic associations.

The increase in maternal lipids as a function of gestational weight gain was estimated from a regression based on measurements in women between 0 and 36 weeks of pregnancy published by Butte et al. (2003):

Maternal lipid gain (kg)*_i_* = 0.675 *×* Gestational weight gain (kg)*_i_* – 3.89 + Residual lipid gain (kg)*_i_*, [3]

where the residual lipid gain (the gain in lipids not explained by gestational weight gain alone) was sampled from a normal distribution centered around 0 and an SD of 2.69 kg truncated at ± 2 SD, and *i* indexes “subject.” We assumed this relationship to be valid beyond 36 weeks of pregnancy. Birth weight was simulated based on gestational weight gain data from 48,743 mother–infant dyads in the Collaborative Perinatal Project (Niswander and Gordon 1972):

Birth weight (kg)*_i_* = 2.982 + 0.02262 *×* Gestational weight gain (kg)*_i_* + Residual birth weight (kg)*_i_*, [4]

where the residual birth weight (the birth weight not explained by gestational weight gain alone) was sampled from a normal distribution with a mean of 0 and an SD of 0.49 kg with a minimum of –2.82 and a maximum of 2.23.

We used acslX (Aegis Technologies Group, Inc., Huntsville, AL, USA) for Monte Carlo simulations. Parameters calculated using this approach in Monte Carlo simulations accurately replicated previously reported Pearson’s correlation coefficients between gestational weight gain and maternal lipid gain [simulated data = 0.79; Butte et al. (2003) = 0.76] and birth weight (simulated data = 0.21; Collaborative Perinatal Project = 0.21).

*Pharmacokinetic model evaluation*. We evaluated the pharmacokinetic model by comparing simulated blood PCB-153 levels to serial blood PCB measurements in 10 pregnant women from another study population who provided blood for PCB measurements on multiple occasions between the 9th and the 37th week of pregnancy (Glynn et al. 2011). We performed a total of 5,000 Monte Carlo simulations for each woman of the Glynn et al. (2011) study by incorporating individual-specific age at delivery, prepregnancy body weight and gestational weight gain, and randomly sampling other physiologic parameters from distributions presented in Table 1. For each individual woman, we calculated the daily PCB-153 dose (micrograms per kilogram body weight) so the simulated plasma level matched that measured in the first blood sample drawn at 9–13 weeks of gestation. The resulting simulated plasma PCB-153 profiles were visually compared with subsequent plasma measurements.

In addition, we ran a Pearson correlation analysis between simulated gestational weight gain and maternal plasma PCB-153 levels and compared the coefficient to the one calculated using data on 2,745 pregnant women from the Collaborative Perinatal Project whose blood was sampled at around 7 months of pregnancy (Longnecker et al. 2005). A similar correlation coefficient in the Monte Carlo simulations and in the Collaborative Perinatal Project data would indicate a good representation of the impact of gestational weight gain on plasma PCB-153 during pregnancy.

*Statistical analyses*. From each Monte Carlo simulation, estimated plasma PCB-153 levels were compiled for each gestational month and at delivery. Simulated gestational weight gain and birth weight data were also compiled for subsequent statistical analyses. Data from the Monte Carlo simulations were first analyzed by linear regression without adjusting for other covariates:

Birth weight (g) = α + β *×* Plasma PCB-153 level (μg/L) + ε, [5]

where α is the intercept, β is the regression coefficient, and ε is the error term. We performed a total of 250,000 Monte Carlo simulations to estimate the value of β in the regression model of Equation 5. We also fit a multivariable linear regression model to determine whether adjusting for gestational weight gain influenced the association between simulated PCB-153 levels and birth weight:

Birth weight (g) = α + β_1_ *×* Plasma PCB-153 level (μg/L) + β_2_ *×* Gestational weight gain (kg) + ε. [6]

Statistical analyses were carried out using IBM SPSS Statistics 20 (IBM, Armonk, NY, USA).

*Simulations of 1,1-dichloro-2,2-bis(*p*-chlorophenyl)ethylene (*p,p´*-DDE)*. In addition to PCB-153, Govarts et al. (2012) evaluated the association between cord serum *p,p´*-DDE levels and birth weight. We also performed Monte Carlo simulations for *p,p´*-DDE to evaluate the influence of gestational weight gain on the association between cord plasma *p,p´*-DDE and birth weight. We used the same pharmacokinetic model and parameters as we used for PCB-153 (Table 1), except for half-life [mean ± SD = 13 ± 2 years (assuming a 15% coefficient of variation) (Wolff et al. 2000)] and maternal plasma level at conception (mean ± SD = 2.099 ± 1.633 μg/L).

*Sensitivity analyses*. We performed sensitivity analyses to evaluate the impact of certain model assumptions on the results of our study. First, our calculation of maternal lipid gain as a function of gestational weight gain was based on the regression published by Butte et al. (2003). In their study, Butte et al. (2003) used equations derived from experiments on nonpregnant subjects (Fuller et al. 1992) and measurements taken both before (bone mineral content) and during their pregnancy (body volume, total body water, and body weight) to estimate maternal lipids at different times during pregnancy. Because measurements used to estimate maternal lipids during pregnancy may conflate both the mother and the fetus, it is possible that our estimations of maternal lipids during pregnancy were slightly overestimated. To evaluate how this may affect our findings, we also performed Monte Carlo simulations where estimated fetal lipids were subtracted from estimated maternal lipids.

Second, we considered only the correlations between gestational weight gain and the increase in maternal lipids and birth weight. Correlations between other parameters may also influence the association between PCB-153 exposure and birth weight. Of particular interest are the associations between prepregnancy body weight and both gestational weight gain and birth weight. We ran a sensitivity analysis with these additional associations (based on our analysis of the Collaborative Perinatal Project data) to quantify their impact on our findings. Gestational weight gain was first derived from prepregnancy body weight:

Gestational weight gain (kg)*_i_* = 11.567 – 0.218 *×* Prepregnancy body weight (kg)*_i_* + Residual gestational weight gain (kg)*_i_*, [7]

where the residual gestational weight gain was sampled from a normal distribution with a mean ± SD of 0.0 ± 4.7 kg. Then birth weight was simulated based on both gestational weight gain and prepregnancy body weight:

Birth weight (kg)*_i_* = 2.383 + 0.009 *×* Prepregnancy body weight (kg)*_i_* + 0.026 *×* Gestational weight gain (kg)*_i_* + Residual birth weight (kg)*_i_*, [8]

where the residual birth weight was sampled from a normal distribution with a mean ± SD of 0.0 ± 0.5 kg.

## Results

**Figure 3:**
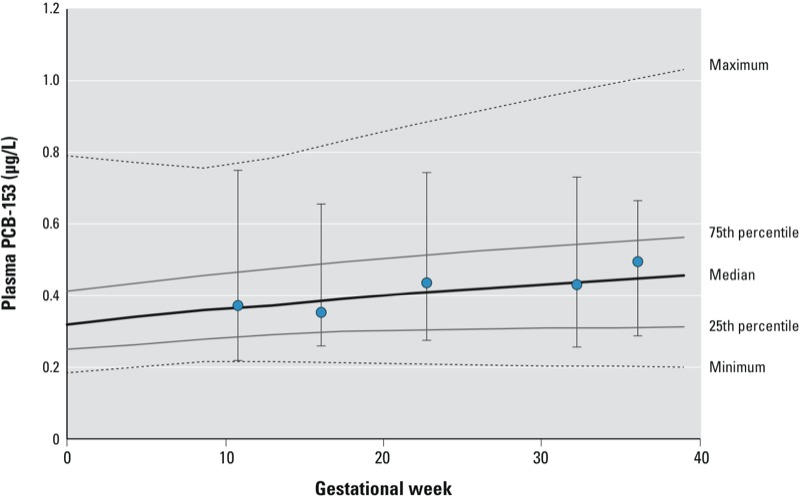
Comparison of simulated plasma PCB-153 levels versus serial plasma measurements in 10 pregnant women from another study population. The lines represent the minimum, 25th percentile, median, 75th percentile, and maximum plasma PCB-153 concentrations predicted from 5,000 Monte Carlo simulations that were carried out for each of the 10 individuals included by Glynn et al. (2011) based on the first PCB-153 measurement at 9–13 weeks of gestation. Circles represent the median measured PCB-153 levels at different times, and error bars represent the minimum and maximum measured plasma PCB-153 levels among the 10 women included by Glynn et al. (2011).

*Pharmacokinetic model evaluation*. Simulated plasma PCB-153 levels were in good agreement with median values measured during pregnancy among the 10 women included in the Glynn et al. (2011) study (Figure 3). Levels reported by Glynn et al. (2011) increased from a median of 0.37 μg/L at 9–13 weeks of gestation to 0.48 μg/L at 35–36 weeks. Similarly, simulated plasma levels at 35 weeks had a median of 0.45 μg/L. The Pearson correlation coefficient between simulated gestational weight gain and maternal plasma PCB-153 level at 7 months of pregnancy (*r* = –0.15) was comparable to the correlation estimated using data from the Collaborative Perinatal Project (*r* = –0.13), indicating a good representation of the impact of gestational weight gain on plasma PCB-153 during pregnancy.

*Association between simulated plasma PCB-153 and birth weight*. The crude association between simulated plasma PCB-153 levels and birth weight increased in strength during pregnancy (Table 2). Maternal plasma PCB-153 levels at the beginning of pregnancy, when gestational weight gain has little influence on the dilution of PCBs, showed no association with birth weight. However, an association appeared with increasing gestational weeks that was strongest at delivery, resulting in an estimated reduction of 30 g in birth weight (95% CI: –33, –27 g) with each 1-μg/L increase in PCB-153 in maternal plasma. A 1-μg/L increase in PCB-153 in cord plasma was associated with a 118-g decrease in mean birth weight (95% CI: –129, –106 g), which was slightly smaller than the 150-g decrease (95% CI: –240, –50 g) estimated based on the meta-analysis by Govarts et al (2012). In our analyses with *p,p´*-DDE, we estimated a 31-g (95% CI: –34, –28 g) decrease in birth weight for each 1-μg/L increase in cord plasma level compared with the 7-g decrease (95% CI: –18, 4 g) reported by Govarts et al (2012).

**Table 2:** Changes in birth weight (g) associated with a 1-μg/L increase in simulated plasma PCB‑153 levels from 250,000 Monte Carlo simulations.

Time	β (95% CI)^*a*^
Maternal plasma
Conception	3.75 (–1.03, 8.53)
1st month	–2.54 (–7.01, 1.94)
2nd month	–7.79 (–11.99, –3.60)
3rd month	–12.22 (–16.17, –8.27)
4th month	–16.06 (–19.79, –12.34)
5th month	–19.49 (–23.01, –15.97)
6th month	–22.49 (–25.83, –19.16)
7th month	–25.24 (–28.42, –22.07)
8th month	–27.71 (–30.73, –24.69)
Delivery	–29.75 (–32.62, –26.88)
Cord plasma
Delivery	–117.55 (–129.31, –105.79)
^***a***^β and 95% CI were estimated from linear regression models unadjusted for gestational weight gain.	

The association was similar in sensitivity analyses when the estimated volume of fetal lipids was subtracted from the estimated volume of maternal lipids (101-g decrease in birth weight; 95% CI: –112, –90 g) and when we accounted for the correlation between prepregnancy body weight and gestational weight gain and birth weight (125-g decrease in birth weight; 95% CI: –137, –112 g). When we adjusted for gestational weight gain (Equation 6), the associations were greatly reduced: We estimated a 2-g decrease (95% CI: –4, 1 g) in birth weight with each 1-μg/L increase in PCB-153 in maternal plasma at delivery, and a 6-g decrease (95% CI: –18, 6 g) with each 1-μg/L increase in PCB-153 in cord plasma. The effect estimate remained unchanged in the regression model of cord plasma PCB-153 and birth weight adjusting for age at delivery (continuous), prepregnancy body weight (continuous), and child sex (dichotomous) (β: 118 g, 95% CI: –130, –106 g).

## Discussion

In this study, we assessed whether the association between cord PCB-153 levels and birth weight reported in a recent meta-analysis (Govarts et al. 2012) might be attributable to the influence of gestational weight gain on both PCB-153 levels and birth weight. Simulated data generated using a pharmacokinetic model suggested that the association is strongly confounded by gestational weight gain, implying that associations between prenatal PCB levels and birth weight that are not adjusted for gestational weight gain may be biased away from the null.

The negative association between simulated maternal plasma PCB-153 levels and birth weight increased with gestational weeks. The strongest association was at delivery, when weight gain was the greatest and consequently had the largest estimated effect on simulated PCB levels. In addition, the negative association with birth weight was larger for cord plasma levels than maternal levels at delivery. This is attributable to the narrower range of PCB concentrations in cord blood: A 1-unit change in cord plasma levels represents a greater relative increase in exposure than a 1-unit change in maternal plasma levels. Interestingly, Govarts et al. (2012) also found that the estimated association between PCB levels and birth weight was larger in the studies that measured PCB levels in cord blood than in studies where cord serum levels were estimated from maternal serum levels using conversion factors. The effect estimate observed in the present study was comparable, although smaller, to the summary estimate reported in the meta-analysis by Govarts et al. (2012), which was not adjusted for gestational weight gain. Their secondary analyses in cohorts where information on gestational weight gain was available revealed that adjustment for this confounder had little influence on the effect estimate. In contrast, adjustment for gestational weight gain greatly reduced the magnitude of the negative association in our study. We note that because gestational weight gain is not a precise surrogate measure of the increased maternal lipids during pregnancy, adjustment for it would not be expected to completely attenuate an apparent association between PCB-153 levels and birth weight. In addition, we would expect that adjustment would be more incomplete when gestational weight gain is classified based on mothers’ recall rather than measured weight gain.

Although our study focused on PCB-153, confounding by gestational weight gain is expected to occur for other lipophilic persistent organic pollutants, such as *p,p´*-DDE, and polybrominated diphenyl ethers (PBDEs). For example, PBDEs have also been associated with decreased birth weight (Harley et al. 2011). In their study, Harley et al. (2011) reported a 140-g decrease (95% CI: –254, –26 g) in birth weight for each 10-fold increase in the sum of PBDEs in maternal serum at about 26 weeks of gestation. When they adjusted for gestational weight gain, this association was reduced to a 99-g decrease (95% CI: –216, 19 g) in birth weight. The remaining association, although nonsignificant, could indicate that the relationship between PBDEs and birth weight is not attributable simply to gestational weight gain. Govarts et al. (2012) reported a 7-g decrease (95% CI: –18, 4 g) in birth weight for each 1-μg/L increase in cord plasma *p,p´*-DDE. The authors suggested that the weaker association between *p,p´*-DDE and birth weight compared with PCB-153 is evidence against gestational weight gain being the underlying cause of the negative association between PCB-153 and birth weight. When we used our pharmacokinetic methodology with *p,p´*-DDE, we also estimated a weaker association than that obtained in our analyses with PCB-153 (*p,p´*-DDE, β: –31, 95% CI: –34, –28, compared with PCB-153, β: –118, 95% CI: –129, –106). Because we have found similar results solely on the basis of pharmacokinetics, it is possible that the reported difference in strength between PCB-153 and *p,p´*-DDE is attributable to noncausal factors (e.g., the difference in the range of serum concentrations).

Limitations of our study include the strong dependence of results on our assumptions regarding the quantitative relationships between gestational weight gain and maternal lipid gain and birth weight in the Monte Carlo simulations. Also, the regression of maternal lipid gain as a function of gestational weight gain estimated by Butte et al. (2003) was based on measurements in a sample of just 63 women. The effect estimates and standard error may be different on a population level, which would influence the strength of the association between gestational weight gain and PCB-153 levels. Nonetheless, the correlation between gestational weight gain and maternal plasma PCB-153 levels was similar for our simulated data and the epidemiologic data from the Collaborative Perinatal Project, suggesting that this phenomenon was captured accurately by the model. Finally, the regression between gestational weight gain and birth weight in the Collaborative Perinatal Project may not be generalizable to the contemporary European population studied by Govarts et al. (2012).

## Conclusions

Our study suggests that the association between prenatal levels of PCBs and birth weight may be strongly confounded by the effect of gestational weight gain on both blood PCB levels and birth weight. Although controlling for gestational weight gain in epidemiologic studies of associations between prenatal exposures to lipophilic persistent organic pollutants such as PCBs and birth weight is important, because gestational weight gain is an imprecise measure of the increased maternal lipid compartment during pregnancy, such adjustment will not completely remove the bias. For other epidemiologic associations between pollutants and health outcomes that may be attributable partly to pharmacokinetics, an approach such as the one used here may provide quantitative insight into its contribution to the relationship.

## Supplemental Material

(156 KB) PDF
