# Erratum: “Is the Relationship between Prenatal Exposure to PCB-153 and Decreased Birth Weight Attributable to Pharmacokinetics?”

**DOI:** 10.1289/ehp.122-A93

**Published:** 2014-04-01

In “Is the Relationship between Prenatal Exposure to PCB-153 and Decreased Birth Weight Attributable to Pharmacokinetics?” by Verner et al. [Environ Health Perspect 121:1219–1224; http://dx.doi.org/10.1289/ehp.1206457 (2013)], the disclaimer was inadvertently omitted from the Competing Financial Interest Declaration. The complete statement appears below.

This study was supported by the American Chemistry Council (ACC) Long-Range Research Initiative and the Intramural Research Program of the National Institute of Environmental Health Sciences, National Institutes of Health. M.-A.V. conducted this study as a consultant for the Hamner Institutes for Health Sciences, an independent nonprofit organization. M.P.L. received no compensation from the ACC. The authors certify that their freedom to design, conduct, interpret, and publish research was not compromised by any sponsor.

*EHP* regrets the error.

