# Supplementary material for: Is the Relationship between Prenatal Exposure to PCB-153 and Decreased Birth Weight Attributable to Pharmacokinetics?
Source: Environ Health Perspect. 2013 Aug 9;121(10):1219–24. doi: 10.1289/ehp.1206457 (PMC3801461; doi:10.1289/ehp.1206457)
Supplement: (156 KB) PDF [file ehp.1206457.s001.pdf]

SUPPLEMENTAL MATERIAL

Is the Relationship between Prenatal Exposure to PCB-153 and Decreased Birth Weight Attributable to Pharmacokinetics?

Marc-André Verner, Robin McDougall, Anders Glynn, Melvin E Andersen, Harvey J Clewell 3rd, Matthew P Longnecker

Table of contents

Model code (CSL file) 2
Model script (M file)..6

! MODEL CODE (CSL FILE)
! Software: acslX (Aegis Technologies Group, Inc., Huntsville, AL, USA)

```
PROGRAM

!***********************************!
!***********************************!
!**   PCB-153 toxicokinetic model **!
!**   Coded by Marc-Andre Verner  **!
!**                               **!
!**   Units                       **!
!**   Time: hours                 **!
!**   Volumes: L                  **!
!**   Weights: kg                 **!
!**   Quantities: ug              **!
!***********************************!
!***********************************!


INITIAL    ! Start of initial section

!*******************************************************************************
! Individual information

! Maternal variables
CONSTANT AGE_DELIVERY                 = 25.00   ! Age of mother at delivery (years)
CONSTANT PREPREGNANCY_BW              = 60.0    ! Pre-pregnancy body weight (kg)
CONSTANT GWG                          = 12.5    ! Gestational weight gain (kg)
CONSTANT MULTIPLIER_MATERNAL_LIPIDS   = 1.0     ! Multiplier for maternal body lipids
CONSTANT MULTIPLIER_PLASMA_LIPIDS     = 1.0     ! Multiplier for lipids in maternal plasma
CONSTANT LIPID_GAIN                   = 5.0     ! Lipid gain during pregnancy (kg)
CONSTANT HALF_LIFE_PCB153             = 14.4    ! PCB-153 half-life (years)

! Fetal variables
CONSTANT SEX                          = 0       ! Sex (0=female, 1=male)
CONSTANT GESTATIONAL_AGE              = 0.75    ! Gestational age at birth (years)
CONSTANT BIRTHWEIGHT                  = 3.4     ! Birthweight (kg)
CONSTANT MULTIPLIER_FETAL_LIPIDS      = 1.0     ! Multiplier for fetal body lipids
CONSTANT CORD_PLASMA_LIPIDS           = 1.83    ! Lipids in cord (g/L)

! Dosing variable
CONSTANT DOSE                         = 0.01    ! Daily oral dose (ug/kg)

! Sample variables for dose optimization using the script
CONSTANT PCB153_LEVEL                 = 50.0    ! Maternal PCB-153 level at sample (ug/kg lipid)
CONSTANT MOTHER_AGE_SAMPLE            = 24.25   ! Age of mother at sample (years)

END ! End of initial section


DYNAMIC ! Start of dynamic sections

!*******************************************************************************
! Length of simulations

TSTOPage = AGE_delivery           ! Length of simulation / mother's age (yrs)
TSTOP = 24*365*TSTOPage           ! Length of simulation (hrs)
POINTS = 300                      ! Number of points in plot
schedule stopthesim .at. TSTOP    ! Schedule end of simulation
discrete stopthesim               ! When simulation reaches stopthesim event...
termt(.true.)                     ! ...terminate simulation
end                               ! End of discrete event


!*******************************************************************************
! Simulation parameters

ALGORITHM IALG = 15        ! Use CVODE algorithm
MAXTERVAL MAXT = 100000    ! Maximum interval for integrations
MINTERVAL MINT = 0.01      ! Minimal interval for integrations
CINTERVAL CINT = 24        ! Communication interval


DERIVATIVE    ! Start of DERIVATIVE SECTION

!*******************************************************************************
! Time points to record PCB-153 levels

Age_conception = AGE_Delivery-GESTATIONAL_AGE  ! Age at conception (years)
```

```
Age_c_mom_1    = Age_conception+0.083          ! Age at 1 month of pregnancy (years)
Age_c_mom_2    = Age_conception+0.167          ! Age at 2 months of pregnancy (years)
Age_c_mom_3    = Age_conception+0.250          ! Age at 3 months of pregnancy (years)
Age_c_mom_4    = Age_conception+0.333          ! Age at 4 months of pregnancy (years)
Age_c_mom_5    = Age_conception+0.417          ! Age at 5 months of pregnancy (years)
Age_c_mom_6    = Age_conception+0.500          ! Age at 6 months of pregnancy (years)
Age_c_mom_7    = Age_conception+0.583          ! Age at 7 months of pregnancy (years)
Age_c_mom_8    = Age_conception+0.667          ! Age at 8 months of pregnancy (years)
Age_c_mom_9    = Age_conception+0.749          ! Age at 9 months of pregnancy (years)


!************************************************************************************
! Basal body weight (not taking pregnancy weight changes into account)
! Curves are based on 50th percentile charts and adjusted for individual measurements

! Mother
table table_bw_mother,1,10/0.0, 0.5, 1.0, 2.0, 5.0, 10.0, 13.0, 16.0, 20.0, 100.0, &
                           3.4, 7.2, 9.5, 12.1, 18.0, 33.0, 46.0, 54.0, 58.0, 58.0/
Diff_profile = (1-Switch_conception) &
               *(AGE*(PREPREGNANCY_BW-table_bw_mother(Age_conception))/Age_conception) &
               +Switch_conception*(PREPREGNANCY_BW-table_bw_mother(Age_conception))
BW = Diff_profile + table_bw_mother(Age)

! Fetus
table table_fetal_weight,1,7/0.0, 0.217, 0.353, 0.463, 0.551, 0.639, 0.768, &
                             0.0, 0.0, 0.26, 0.69, 1.25, 2.02, 3.28/
NORM_BIRTHWEIGHT_FOR_GA   = table_fetal_weight(GESTATIONAL_AGE)
RATIO_BIRTHWEIGHT_NORM    = BIRTHWEIGHT/NORM_BIRTHWEIGHT_FOR_GA
FETAL_WEIGHT              = (SWITCH_CONCEPTION-SWITCH_DELIVERY) &
                            *RATIO_BIRTHWEIGHT_NORM*table_fetal_weight(FETAL_AGE)


!************************************************************************************
! BODY FAT

! Mother
table bodyfat_mother,1,17/0.0,0.08,0.17,0.25,0.33,0.5,1,2,4,6,8,10,12,16,29,39,69, &
                          0.15,0.16,0.21,0.24,0.25,0.26,0.24,0.20,0.17,0.16,0.17, &
                          0.19,0.23,0.26,0.29,0.31,0.41/
SLOPE_GAIN_PREGNANCY = (SWITCH_CONCEPTION-SWITCH_DELIVERY) &
                       *(LIPID_GAIN*(AGE-AGE_CONCEPTION)/GESTATIONAL_AGE)
MATERNAL_LIPIDS      = MULTIPLIER_MATERNAL_LIPIDS*(BW*bodyfat_mother(age)) &
                       +SLOPE_GAIN_PREGNANCY
table plasma_lipids,1,2/0.0, 0.75, 4.4, 7.9/
MATERNAL_PLASMA_LIPIDS = MULTIPLIER_PLASMA_LIPIDS*plasma_lipids(FETAL_AGE)

! Fetus
FETAL_LIPIDS  = MULTIPLIER_FETAL_LIPIDS &
                *((SWITCH_CONCEPTION-SWITCH_DELIVERY) &
                *FETAL_WEIGHT*(sex*0.14+(1-sex)*0.15)+0.001)


!************************************************************************************
! Elimination

HALF_LIFE  = HALF_LIFE_PCB153*365*24     ! PCB-153 Half-life (hours)


!************************************************************************************
! Rates of transfer

RAO                    = BW*DOSE/24                                     ! PCB-153 intake rate (ug/h)
ELIMINATION            = A_MOTHER*log(2)/HALF_LIFE                      ! Elimination rate
MOTHER_FETUS_TRANSFER = (SWITCH_CONCEPTION-SWITCH_DELIVERY)*(C_MOTHER) ! Mother-fetus diffusion
FETUS_MOTHER_TRANSFER = (SWITCH_CONCEPTION-SWITCH_DELIVERY)*(C_FETUS)  ! Fetus-mother diffusion


!************************************************************************************
! Age

RAGE      = 1/24./365.                            ! Rate of age increase (years/h)
AGE       = INTEG(RAGE, 0.)                       ! Age of mother (years)
FETAL_AGE = SWITCH_CONCEPTION*(AGE-AGE_CONCEPTION)  ! Age of fetus (years)


!************************************************************************************
! Discrete events

! Set initial values to zero
initial
SWITCH_CONCEPTION      = 0   ! I/O Switch for beginning of pregnancy
```

```
SWITCH_DELIVERY        = 0   ! I/O Switch for delivery
C_MOTHER_3RULE_LIPIDS  = 0   ! PCB-153 level in maternal lipids at sample (ug/kg lipid)
C_MONTH_0_LIPIDS       = 0   ! PCB-153 in maternal lipids - conception (ug/kg lipid)
C_MONTH_1_LIPIDS       = 0   ! PCB-153 in maternal lipids - 1st month of pregnancy (ug/kg lipid)
C_MONTH_2_LIPIDS       = 0   ! PCB-153 in maternal lipids - 2nd month of pregnancy (ug/kg lipid)
C_MONTH_3_LIPIDS       = 0   ! PCB-153 in maternal lipids - 3rd month of pregnancy (ug/kg lipid)
C_MONTH_4_LIPIDS       = 0   ! PCB-153 in maternal lipids - 4th month of pregnancy (ug/kg lipid)
C_MONTH_5_LIPIDS       = 0   ! PCB-153 in maternal lipids - 5th month of pregnancy (ug/kg lipid)
C_MONTH_6_LIPIDS       = 0   ! PCB-153 in maternal lipids - 6th month of pregnancy (ug/kg lipid)
C_MONTH_7_LIPIDS       = 0   ! PCB-153 in maternal lipids - 7th month of pregnancy (ug/kg lipid)
C_MONTH_8_LIPIDS       = 0   ! PCB-153 in maternal lipids - 8th month of pregnancy (ug/kg lipid)
C_MONTH_9_LIPIDS       = 0   ! PCB-153 in maternal lipids - delivery (ug/kg lipid)
C_CORD_LIPIDS          = 0   ! PCB-153 in cord plasma lipids - delivery (ug/kg lipid)
C_MOTHER_3RULE_PLASMA  = 0   ! PCB-153 in maternal plasma at sample (ug/L)
C_MONTH_0_PLASMA       = 0   ! PCB-153 in maternal plasma - conception (ug/L)
C_MONTH_1_PLASMA       = 0   ! PCB-153 in maternal plasma - 1st month of pregnancy (ug/L)
C_MONTH_2_PLASMA       = 0   ! PCB-153 in maternal plasma - 2nd month of pregnancy (ug/L)
C_MONTH_3_PLASMA       = 0   ! PCB-153 in maternal plasma - 3rd month of pregnancy (ug/L)
C_MONTH_4_PLASMA       = 0   ! PCB-153 in maternal plasma - 4th month of pregnancy (ug/L)
C_MONTH_5_PLASMA       = 0   ! PCB-153 in maternal plasma - 5th month of pregnancy (ug/L)
C_MONTH_6_PLASMA       = 0   ! PCB-153 in maternal plasma - 6th month of pregnancy (ug/L)
C_MONTH_7_PLASMA       = 0   ! PCB-153 in maternal plasma - 7th month of pregnancy (ug/L)
C_MONTH_8_PLASMA       = 0   ! PCB-153 in maternal plasma - 8th month of pregnancy (ug/L)
C_MONTH_9_PLASMA       = 0   ! PCB-153 in maternal plasma - delivery (ug/L)
C_CORD_PLASMA          = 0   ! PCB-153 in cord plasma - delivery (ug/L)
end

! Update values at discrete events
schedule mothersample .xn. (MOTHER_AGE_SAMPLE-AGE)
discrete mothersample
  C_MOTHER_3RULE_PLASMA = C_MOTHER_P
  C_MOTHER_3RULE_LIPIDS = C_MOTHER
End
schedule c_mom_month0 .xn. (Age_conception-AGE)
discrete c_mom_month0
       SWITCH_CONCEPTION = 1
       C_MONTH_0_PLASMA  = C_MOTHER_P
       C_MONTH_0_LIPIDS  = C_MOTHER
End
schedule delivery .xn. (AGE_delivery-AGE)
discrete delivery
       SWITCH_DELIVERY = 1
End
schedule c_mom_month1 .xn. (Age_c_mom_1-AGE)
discrete c_mom_month1
       C_MONTH_1_PLASMA = C_MOTHER_P
       C_MONTH_1_LIPIDS = C_MOTHER
End
schedule c_mom_month2 .xn. (Age_c_mom_2-AGE)
discrete c_mom_month2
       C_MONTH_2_PLASMA = C_MOTHER_P
       C_MONTH_2_LIPIDS = C_MOTHER
End
schedule c_mom_month3 .xn. (Age_c_mom_3-AGE)
discrete c_mom_month3
       C_MONTH_3_PLASMA = C_MOTHER_P
       C_MONTH_3_LIPIDS = C_MOTHER
End
schedule c_mom_month4 .xn. (Age_c_mom_4-AGE)
discrete c_mom_month4
       C_MONTH_4_PLASMA = C_MOTHER_P
       C_MONTH_4_LIPIDS = C_MOTHER
End
schedule c_mom_month5 .xn. (Age_c_mom_5-AGE)
discrete c_mom_month5
       C_MONTH_5_PLASMA = C_MOTHER_P
       C_MONTH_5_LIPIDS = C_MOTHER
End
schedule c_mom_month6 .xn. (Age_c_mom_6-AGE)
discrete c_mom_month6
       C_MONTH_6_PLASMA = C_MOTHER_P
       C_MONTH_6_LIPIDS = C_MOTHER
End
schedule c_mom_month7 .xn. (Age_c_mom_7-AGE)
discrete c_mom_month7
       C_MONTH_7_PLASMA = C_MOTHER_P
       C_MONTH_7_LIPIDS = C_MOTHER
End
schedule c_mom_month8 .xn. (Age_c_mom_8-AGE)
discrete c_mom_month8
       C_MONTH_8_PLASMA = C_MOTHER_P
```

```
       C_MONTH_8_LIPIDS = C_MOTHER
End
schedule c_mom_month9 .xn. (Age_c_mom_9-AGE)
discrete c_mom_month9
       C_MONTH_9_PLASMA =  C_MOTHER_P
       C_MONTH_9_LIPIDS =  C_MOTHER
  C_CORD_PLASMA    = (C_FETUS/1000)*CORD_PLASMA_LIPIDS
  C_CORD_LIPIDS    =  C_FETUS
End


!*************************************************************************************
! Mass balance differential equations

RA_MOTHER  = RAO-ELIMINATION-MOTHER_FETUS_TRANSFER &
             +FETUS_MOTHER_TRANSFER                       ! Rate PCB-153 in maternal lipids (ug/h)
A_MOTHER   = INTEG(RA_MOTHER, 0.0)                        ! PCB-153 in maternal lipids (ug)
C_MOTHER   = A_MOTHER/MATERNAL_LIPIDS                     ! Concentration in maternal lipids (ug/kg)
C_MOTHER_P = (C_MOTHER/1000)*MATERNAL_PLASMA_LIPIDS       ! Concentration in maternal plasma (ug/L)

RA_FETUS   = MOTHER_FETUS_TRANSFER-FETUS_MOTHER_TRANSFER  ! Rate PCB-153 in fetal lipids (ug/h)
A_FETUS    = INTEG(RA_FETUS, 0.0)                         ! PCB-153 in fetal lipids (ug)
C_FETUS    = A_FETUS/FETAL_LIPIDS                         ! Concentration in fetal lipids (ug/kg)

END ! DERIVATIVE

END ! DYNAMIC

END ! PROGRAM
```

! MODEL SCRIPT (M FILE)
! Software: acslX (Aegis Technologies Group, Inc., Huntsville, AL, USA)

```
%************************************************************%
% Script for the Verner et al. 2013 PCB-Birthweight analyses %
%************************************************************%

prepare @all
save

%**********************%
% Simulation parameters %
%**********************%
CIEITG = 0;
WESITG = 0;
WXDITG = 0;
WEDITG = 0;
CJVITG = 0;
WNDITG = 0;
CINT   = 100000;
MINT   = 0.1;
MAXT   = 100000;
IALG   = 15;


%*******************************************%
% Creates arrays to record simulated data %
%*******************************************%
C_MONTH_0_LIPIDSs  = [];
C_MONTH_1_LIPIDSs  = [];
C_MONTH_2_LIPIDSs  = [];
C_MONTH_3_LIPIDSs  = [];
C_MONTH_4_LIPIDSs  = [];
C_MONTH_5_LIPIDSs  = [];
C_MONTH_6_LIPIDSs  = [];
C_MONTH_7_LIPIDSs  = [];
C_MONTH_8_LIPIDSs  = [];
C_MONTH_9_LIPIDSs  = [];
C_CORD_LIPIDSs     = [];
C_MONTH_0_PLASMAs  = [];
C_MONTH_1_PLASMAs  = [];
C_MONTH_2_PLASMAs  = [];
C_MONTH_3_PLASMAs  = [];
C_MONTH_4_PLASMAs  = [];
C_MONTH_5_PLASMAs  = [];
C_MONTH_6_PLASMAs  = [];
C_MONTH_7_PLASMAs  = [];
C_MONTH_8_PLASMAs  = [];
C_MONTH_9_PLASMAs  = [];
C_CORD_PLASMAs     = [];
doses              = [];
dataMCs            = [];


%*******************************%
% Start of automation script    %
%*******************************%

Num_MC_iterations=250000;  % Number of individuals
seedrnd(123456789);        % Seed

for iteration=[1:1:Num_MC_iterations]

% Display iteration in command window
disp(sprintf("Iteration #%d of %d", iteration, Num_MC_iterations));
disp("---------------------------");

% Parameter distributions and regressions
AGE_DELIVERY               = normrnd(24.3, 6.1, 15.0, 41.0);   % Age at delivery (years)
PREPREGNANCY_BW            = normrnd(59.0, 11.6, 25.0, 163.0); % Pre-pregnancy body weight (kg)
GWG                        = normrnd(10.3, 4.7, 0.0, 44.0);    % Gestational weight gain (kg)
MULTIPLIER_MATERNAL_LIPIDS = normrnd(1.0, 0.16, 0.68, 1.32);   % Maternal body lipids multiplier
MULTIPLIER_PLASMA_LIPIDS   = normrnd(1.0,0.13,0.72,1.34);      % Maternal plasma lipids multiplier
HALF_LIFE_PCB153           = normrnd(14.4, 2.2, 10.0, 18.8);   % PCB-153 half-life (years)
RESID_LIPID_GAIN           = normrnd(0.0, 2.69, -5.4, 5.4);    % Residuals GWG-lipid gain (kg)
LIPID_GAIN                 = 0.675*GWG-3.89+RESID_LIPID_GAIN;  % Lipid gain during pregnancy (kg)
SEX                        = bernrnd(0.5);                     % Sex (Female=0, Male=1)
```

```
MULTIPLIER_FETAL_LIPIDS    = normrnd(1.0, 0.17, 0.66, 1.34);   % Fetal lipids multiplier
CORD_PLASMA_LIPIDS         = normrnd(1.83, 0.24, 1.35, 2.31);  % Lipids in cord plasma (g/L)
RESID_BW                   = normrnd(0.0, 0.49, -2.82, 2.23);  % Residuals birthweight (kg)
BIRTHWEIGHT                = 0.02262*GWG+2.982+RESID_BW;       % Birthweight (kg)
MOTHER_AGE_SAMPLE          = AGE_DELIVERY-0.75 ;               % Age at conception (years)

% Maternal plasma PCB-153 levels at conception
% (to replicate the range of cord serum levels in Govarts et al. [2012])
ASD          = 0.412;                           % Arithmetic standard deviation
AM           = 0.487;                           % Arithmetic mean (ug/L)
sigma        = sqrt(log(1+ASD^2/AM^2));         % Conversion for the lognormal distribution
mu           = log(AM)-0.5*sigma^2;             % Conversion for the lognormal distribution
PCB153_LEVEL = lognrnd(mu, sigma, 0.001, 10.0);  % Maternal plasma level at conception (ug/L)

% Dose no1 to start the daily dose optimization process
DOSE      = 0.01;

% Start first simulation
start @nocallback

% Optimization of daily dose to match sampled PCB-153 levels (PCB153_LEVEL)
rule3 = C_MOTHER_3RULE_PLASMA;
DOSE  = PCB153_LEVEL*0.01/rule3;
doses = [doses; DOSE];

% Start second simulation
start @nocallback

% Record results
C_MONTH_0_LIPIDSs  = C_MONTH_0_LIPIDS;
C_MONTH_1_LIPIDSs  = C_MONTH_1_LIPIDS;
C_MONTH_2_LIPIDSs  = C_MONTH_2_LIPIDS;
C_MONTH_3_LIPIDSs  = C_MONTH_3_LIPIDS;
C_MONTH_4_LIPIDSs  = C_MONTH_4_LIPIDS;
C_MONTH_5_LIPIDSs  = C_MONTH_5_LIPIDS;
C_MONTH_6_LIPIDSs  = C_MONTH_6_LIPIDS;
C_MONTH_7_LIPIDSs  = C_MONTH_7_LIPIDS;
C_MONTH_8_LIPIDSs  = C_MONTH_8_LIPIDS;
C_MONTH_9_LIPIDSs  = C_MONTH_9_LIPIDS;
C_CORD_LIPIDSs     = C_CORD_LIPIDS;
C_MONTH_0_PLASMAs  = C_MONTH_0_PLASMA;
C_MONTH_1_PLASMAs  = C_MONTH_1_PLASMA;
C_MONTH_2_PLASMAs  = C_MONTH_2_PLASMA;
C_MONTH_3_PLASMAs  = C_MONTH_3_PLASMA;
C_MONTH_4_PLASMAs  = C_MONTH_4_PLASMA;
C_MONTH_5_PLASMAs  = C_MONTH_5_PLASMA;
C_MONTH_6_PLASMAs  = C_MONTH_6_PLASMA;
C_MONTH_7_PLASMAs  = C_MONTH_7_PLASMA;
C_MONTH_8_PLASMAs  = C_MONTH_8_PLASMA;
C_MONTH_9_PLASMAs  = C_MONTH_9_PLASMA;
C_CORD_PLASMAs     = C_CORD_PLASMA;
doses = DOSE;

% Compile results in an array
dataMCs = [dataMCs; C_MONTH_0_LIPIDSs C_MONTH_1_LIPIDSs C_MONTH_2_LIPIDSs ...
                    C_MONTH_3_LIPIDSs C_MONTH_4_LIPIDSs C_MONTH_5_LIPIDSs ...
                    C_MONTH_6_LIPIDSs C_MONTH_7_LIPIDSs C_MONTH_8_LIPIDSs ...
                    C_MONTH_9_LIPIDSs C_CORD_LIPIDSs ...
                    C_MONTH_0_PLASMAs C_MONTH_1_PLASMAs C_MONTH_2_PLASMAs ...
                    C_MONTH_3_PLASMAs C_MONTH_4_PLASMAs C_MONTH_5_PLASMAs ...
                    C_MONTH_6_PLASMAs C_MONTH_7_PLASMAs C_MONTH_8_PLASMAs ...
                    C_MONTH_9_PLASMAs C_CORD_PLASMAs ...
                    doses AGE_DELIVERY PREPREGNANCY_BW GWG MULTIPLIER_MATERNAL_LIPIDS ...
                    MULTIPLIER_PLASMA_LIPIDS HALF_LIFE_PCB153 RESID_LIPID_GAIN ...
                    LIPID_GAIN SEX MULTIPLIER_FETAL_LIPIDS CORD_PLASMA_LIPIDS ...
                    RESID_BW BIRTHWEIGHT MOTHER_AGE_SAMPLE];

end %individuals

%*************************************************************************************%
% Command to write results in a .csv file                                             %
%*************************************************************************************%
save dataMCs @file=PCB_BW_results.csv @format=ascii @separator=comma
```
